# Incidence of upper extremity deep vein thrombosis in the retrosternal reconstruction after esophagectomy

**DOI:** 10.1186/s12893-022-01544-9

**Published:** 2022-03-09

**Authors:** Leo Yamada, Motonobu Saito, Hiroya Suzuki, Shotaro Mochizuki, Eisei Endo, Koji Kase, Misato Ito, Hiroshi Nakano, Naoto Yamauchi, Takuro Matsumoto, Akinao Kaneta, Yasuyuki Kanke, Hisashi Onozawa, Hiroyuki Hanayama, Hirokazu Okayama, Shotaro Fujita, Wataru Sakamoto, Yohei Watanabe, Suguru Hayase, Zenichiro Saze, Tomoyuki Momma, Shinji Ohki, Koji Kono

**Affiliations:** 1 Department of Gastrointestinal Tract Surgery, Fukushima Medical University, 1 Hikarigaoka, Fukushima City, 960-1295 Japan; 2 Shirakawa Kosei General Hospital, 2-1 Toyochikamiyajirou, Shirakawa City, 961-0005 Japan

**Keywords:** Upper extremity deep vein thrombosis, Esophagectomy, Retrosternal reconstruction, Central venous catheter, Thromboprophylaxis

## Abstract

**Background:**

Upper extremity deep vein thrombosis (UEDVT) is relatively rare but cannot be negligible because it can cause fatal complications. Although it is reported that the occurrence rate of UEDVT has increased due to central venous catheter (CVC), cancer, and surgical invasion, there is still limited information for esophagectomy. The aim of this study was to evaluate the clinical factors, including CVC placement and thromboprophylaxis approach, as well as retrosternal space’s width as a predictive factor for UEDVT in patients receiving esophagectomy.

**Methods:**

This study included 66 patients who underwent esophagectomy with retrosternal reconstruction using a gastric tube. All patients routinely underwent contrast-enhanced computed tomography (CT) on the 4th postoperative day. Low-molecular-weight-heparin (LMWH) was routinely administered by the 2nd postoperative day. To evaluate retrosternal space’s width, (a) The distance from sternum to brachiocephalic artery and (b) the distance from sternum to vertebra were measured by preoperative CT, and the ratio of (a) to (b) was defined as the width of retrosternal space.

**Results:**

Among all patients, 11 (16.7%) suffered from UEDVT, and none was preoperatively received CVC placement, while 7 were inserted in non-UEDVT cases. Retrosternal space’s width in patients with UEDVT was significantly smaller than that in patients without UEDVT (0.17 vs. 0.26; P < 0.0001). A cutoff value of the width was 0.21, which has high sensitivity (87%) and specificity (82%) for UEDVT prediction, respectively.

**Conclusion:**

The existence of CVC may not affect the development of UEDVT, but preoperative evaluation of retrosternal ratio may predict the occurrence of UEDVT.

## Introduction

Currently, neoadjuvant chemotherapy/chemoradiation followed by curative esophagectomy is a standard therapy for stage II/III advanced esophageal cancer [1]. Since esophagectomy requires an invasive procedure regardless of the open or thoracoscopic approach, it is well-known that esophagectomy revealed a high incidence of postoperative complications with 40–50% of the cases in Japanese NCD database [2]. Complications after esophagectomy include a wide range of events, such as anastomotic leakage, bleeding, conduit ischemia, recurrent laryngeal nerve injury, surgical site infection, intrathoracic abscess, and venous thromboembolism (VTE) [3, 4].

It is generally accepted that VTE is a concerning matter, which has been reported to be 2.9–7.3% and could lead to pulmonary embolism (PE) and other respiratory sequelae [5, 6]. As well as lower extremity deep vein thrombosis (LEDVT), upper extremity deep vein thrombosis (UEDVT), which occurs in the subclavian, axillary, internal jugular, and/or brachial veins, cannot be negligible, accounting for up to 10% of all documented DVTs [7]. Its incidence has increased, and the presence of central venous catheter (CVC) has been described as the most significant risk factor of UEDVT, at least 50%, followed by cancer and major surgery within 30 days. Compared with patients with LEDVT, patients with UEDVT tend to be younger and more common in cancer, and less likely to have acquired or hereditary thrombophilia [7]. The frequency of acute PE, a fatal complication of UEDVT, is approximately 6–36% and 2–5% for recurrence at 12 months, which should be identified in the early phase [7–10]. Therefore, early detection is crucial, and it is worth investigating the usefulness of various diagnostic tools, including Computed Tomography (CT) [7].

In esophageal cancer patients who underwent esophagectomy followed by gastric tube reconstruction, Takahashi et al. reported that the compression of the left brachiocephalic vein by the narrowness of retrosternal space contributes to the occurrence of UEDVT and referred to the significant difference in the likelihood to generate UEDVT by retrosternal reconstruction compared with the posterior mediastinal route. Also, the effectiveness of preoperative CT examination for evaluating the width of retrosternal was revealed as a predictive factor for UEDVT [11]. However, in the previous study, all the participants received CVC before esophagectomy, and it has not been described whether CVC placement increases the UEDVT occurrence. In addition, the efficacy of postoperative thromboprophylaxis, low-molecular-weight heparin (LMWH), has not been estimated.

In the present study, we confirmed the effectiveness of retrosternal space measurement by preoperative CT and evaluated the significance of CVC placement and prophylactic anticoagulant therapy’s efficacy for UEDVT in the esophageal cancer patients with retrosternal reconstruction.

## Patients and methods

### Patients

The present study enrolled 66 consecutive patients with thoracic esophageal carcinoma who underwent either right transthoracic esophagectomy via thoracotomy or thoracoscopic esophagectomy, all with gastric tube reconstruction through a retrosternal route in the Gastrointestinal Tract Surgery, Fukushima Medical University Hospital from January 2016 to November 2020. CVC was not preoperatively placed in most patients, including peripherally inserted central catheters (PICCs). An approximately 4-cm-wide gastric tube preserving the greater omentum was created, and after pulling up the gastric tube, cervical esophagogastrostomy was performed at the left side of the neck. The level of oral side dissection is routinely at the upper thoracic esophagus, which is at the level of the Aortic arch, and straightening of the gastric tube is performed. The anastomosis site is generally at the cranial side of the left brachiocephalic vein and visible from the cervical incision. Retrosternal reconstruction was routinized in our institution and performed in all 66 cases. Preoperative and postoperative clinicopathological data were collected, including the following information: demographics, type of treated diseases, type of surgical procedure, and data from laboratory tests on the 4th postoperative day. Preoperative blood tests and interviews of past medical history were routinely conducted as triage for coagulation disorder. Preoperative CT was routinely performed to screen for abnormalities in the venous system, and the ultrasound examination will be performed when the interviews of past medical history revealed deep vein thrombosis (DVT) and/or the lower extremity varix in addition to the high score of D-dimer. The clinical and pathological staging was based on the 8th edition of the TNM classification [12].

All patients underwent subcutaneous injection of low molecular weight heparin twice a day from the 2nd postoperative evening. Postoperative complications were defined as any complication with Clavien grade ≧ 1 using the Clavien–Dindo classification [13].

The study was approved by the ethics committee of Fukushima Medical University. All patients provided written informed consent. All experiments were carried out in accordance with the approved study plan and relevant guidelines.

### Diagnosis of postoperative UEDVT

**Fig. 1:**
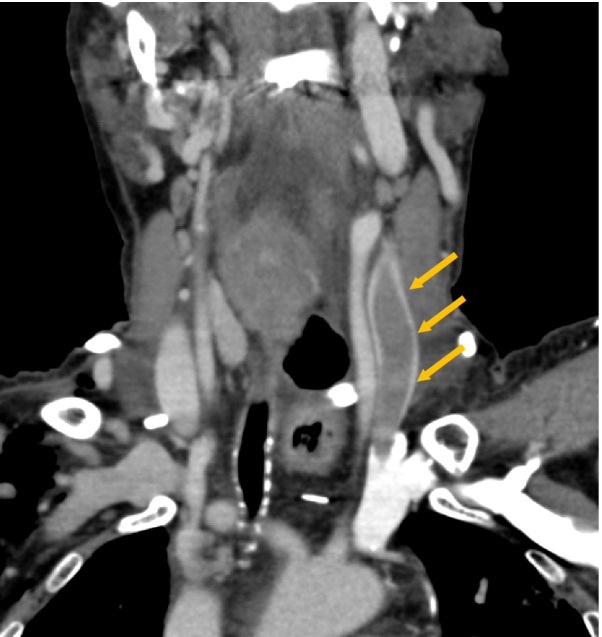
Upper extremity deep vein thrombosis in the left internal jugular vein after esophagectomy with retrosternal reconstruction. Contrast-enhanced CT on the fourth postoperative day and the open arrows indicate an intraluminal filling defect in the left internal jugular vein

To find out any complications, all patients routinely underwent contrast-enhanced computed tomography (CT) from the cervix to the pelvic floor on the 4th postoperative day. UEDVT was defined as an intraluminal filling defect in a brachiocephalic, subclavian, axillary, brachial, internal jugular, or external jugular vein (Fig. 1).

### Measurement of the width of the retrosternal space using preoperative contrast-enhanced computed tomography

**Fig. 2:**
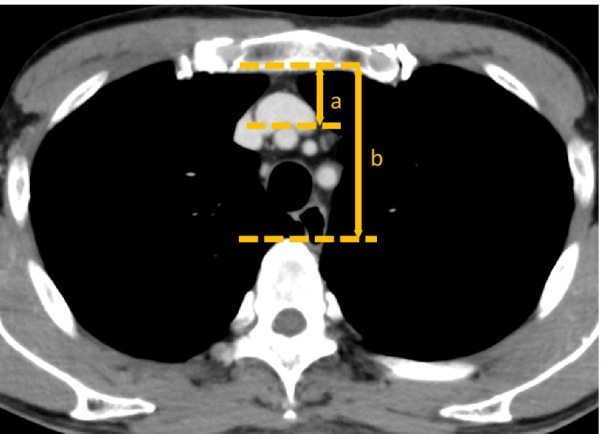
The width of retrosternal space was defined by the ratio of **a** to **b**. **a** the back of the sternum to the ventral part of the brachiocephalic artery. **b** the back of the sternum to the ventral part of the vertebra. To evaluate the retrosternal space, Contrast-enhanced CT was conducted preoperatively within 3 months

To assess the width of the retrosternal space, (a) the distance from the back of the sternum to the ventral part of the brachiocephalic artery and (b) the distance from the back of the sternum to the ventral part of the vertebra were measured using a preoperative axial contrast-enhanced CT image in all the patients. The ratio of (a) to (b) was calculated, and it was defined as the width of the retrosternal space due to the consideration of the difference in individual body shape. The area of the retrosternal space was measured at the level of the left brachiocephalic vein using the preoperative axial contrast-enhanced CT image (Fig. 2) [11].

### Statistical analysis

The data were analyzed using Prism version 9.0.0 (86) (GraphPad Software LLC, San Diego, CA, USA). Continuous data were compared between the two groups using the Mann–Whitney U test or Student’s t test. Categorical data were compared using the Chi-square test. To identify risk factors related to UEDVT, univariate analyses were performed using the Chi-square test. Those variables remaining in the logistics equation at the last step were considered as independent risk factors. P value < 0.05 was considered statistically significant. The optimal cutoff point of the width of the retrosternal space for the prediction of UEDVT was determined so that the Youden index (sensitivity + specificity − 1) would be maximized using receiver operating characteristic (ROC) curve analysis.

## Results

### Incidence and clinical course of UEDVT after esophagectomy

All the esophagectomies were conducted with retrosternal reconstruction. UEDVT developed in 11 of the 66 patients (16.7%). A significant difference was not observed between patients with and without UEDVT regarding clinicopathological factors, including preoperative and operative factors and postoperative complications (Table 1). D-dimer’s elevation did not show specificity by itself, and mean value of D-dimer did not make significant difference between the patients with and without UEDVT (9.91 vs. 7.39, P = 0.4009). Also, there was no significant difference between 2-field and 3-field lymph node dissection (P = 0.46). Among the 11 UEDVT patients (Table 2), 10 patients suffered UEDVTs in the left internal jugular vein and one patient in the left subclavian vein, whereas no UEDVT occurred in the right-side upper extremity deep vein. Small PE without any symptom was detected by the image in 3 cases (27.3%) in the UEDVT patients, which did not result in any severe condition, including respiratory failure or fatalities. For UEDVT patients, prophylaxis LMWH subcutaneous injection was switched to continuous intravenous heparin infusion in optimal dose following guideline [14], and edoxaban was induced when the oral intake started. In all but one case, contrast-enhanced CT was performed three months after esophagectomy. The thrombus had disappeared in 8 of the 10 patients. In the other two cases, contrast-enhanced CT revealed that the thrombus remained, but the anticoagulant therapy was terminated as a chronic thrombosis (Table 2). Regarding follow-up image study after discharge, we routinely perform CT scan within three months and no additional UEDVT occurrences have been observed.

**Table 1:** Clinicopathological characteristics in a total of 66 patients

	Total (n = 66)	UEDVT		P-value^a^
		Positive (n = 11)	Negative (n = 55)	
Age-year				
Mean (range)		63.2 (51–76)	66.8 (41–83)	0.18
Gender (%)				
Male	51	10 (90.9)	41 (74.5)	0.43
Female	15	1 (9.1)	14 (25.5)	
BMI				
Mean (range)		22.5 (18.67–27)	20.9 (15–27.9)	0.087
Smoking history (%)				
Yes		11 (100)	43 (78.2)	0.087
No		0 (0)	12 (21.8)	
Preoperative co morbidity (%)				
Hypertension	30	6 (54.6)	24 (43.6)	0.51
Diabetes mellitus	7	0 (0)	7 (12.7)	0.21
Anti-coagulate drug	2	0 (0)	2 (3.64)	0.52
Respiratory disorder	8	0 (0)	8 (14.6)	0.18
Tumor location (%)				
Ut	10	3 (27.3)	7 (12.7)	0.93
Mt	38	6 (54.5)	32 (58.2)	
Lt	18	2 (18.2)	16 (29.1)	
pStage (%)				
I		4 (36.4)	26 (47.3)	0.187
II		4 (36.4)	6 (10.9)	
III		2 (18.2)	18 (32.7)	
IV		1 (9)	5 (9.1)	
Neoadjuvant therapy (%)				
Yes		5 (45.5)	35 (63.6)	0.25
No		6 (54.5)	20 (36.4)	
Operative approach (%)				
Right transthoracic		7 (63.6)	24 (43.6)	0.23
Thoracoscopic		4 (36.4)	31 (56.4)	
Lymph node dissection (%)				
3-field		10 (90.9)	45 (81.8)	0.46
2-field		1 (9.1)	10 (18.2)	
Operation time, median value (range) (min)	494.9	482.9 (417–601)	497.3 (355–732)	0.60
Blood loss, median value (range) (ml)	283.7	271 (60–905)	286.2 (10–2710)	0.76
Clavien–Dindo Grade				
3b ≦		0 (0)	3 (5.5)	0.43
3a ≧		11 (100)	52 (94.6)	
R0		10 (90.9)	49 (89.1)	0.86
R1/2		1 (9.1)	6 (10.9)	

^a^P values were calculated by Mann–Whitney U test or λ^2^ exact test

**Table 2:** Summary of 11 patients developing UEDVT after esophagectomy

No.	Age	Sex	Location of UEDVT	PE	Symptom	Treatment	Clinical course
1	52	F	Left Internal Jugular	+	None	Heparin → Edoxaban	Disappeared
2	76	M	Left Internal Jugular		None	Heparin → Edoxaban	Disappeared
3	65	M	Left Internal Jugular		None	Heparin → Edoxaban	Disappeared
4	62	M	Left Internal Jugular	+	None	Heparin → Edoxaban	Disappeared
5	64	M	Left Internal Jugular		None	Heparin → Edoxaban	Disappeared
6	65	M	Left Internal Jugular		None	Heparin → Edoxaban	Disappeared
7	62	M	Left Internal Jugular		None	Heparin → Edoxaban	Remaining
8	59	M	Left Internal Jugular		None	Heparin → Edoxaban	Disappeared
9	51	M	Left Internal Jugular		None	Heparin → Edoxaban	Disappeared
10	71	M	Left Internal Jugular		None	Heparin → Edoxaban	Remaining
11	68	M	Left clavicular	+	None	Heparin → Edoxaban	Disappeared

### Width of retrosternal space as a preoperative risk factor for UEDVT after retrosternal reconstruction

To preoperatively estimate the risk of UEDVT in patients with retrosternal reconstruction, we evaluated the width of the retrosternal space. The width of the retrosternal space in patients with UEDVT was significantly smaller than that in patients without UEDVT (0.17 vs. 0.26; P < 0.0001). A cutoff value of 0.21 [AUC: 0.92 (95% CI: 0.84–0.99)] was established so that the Youden index (sensitivity + specificity − 1) would be maximized. Using this cutoff value, UEDVT was detected with a sensitivity of 87% and a specificity of 82% (Table 3). This result indicated the importance of evaluating the retrosternal space as a predictive risk factor before esophagectomy.

**Table 3:** Association between UEDVT and the width of the retrosternal space

	With UEDVT (n = 11)	Without UEDVT (n = 55)	P value
Retrosternal ratio			
< 0.21	9	7	< 0.0001
≧0.21	2	48	

Retrosternal ratio—Ratio of the distance from the back of the sternum to the ventral part of brachiocephalic artery and the distance from the back of the sternum to the ventral part of the vertebra

### Correlation between UEDVT and CVC after esophagectomy with retrosternal reconstruction

Among UEDVT cases, none was preoperatively received CVC placement, while 7 were inserted in non-UEDVT cases (n = 55), and there was no correlation between UEDVT and CVC placement (Table 4, P = 0.21). LMWH was routinely administered twice a day on the 2nd postoperative day as postoperative thromboprophylaxis in all the cases. Also, there was no significant difference in inflammatory response, Albumin, Platelet, and D-dimer between patients with and without UEDVT (Table 4).

**Table 4:** Correlation between UEDVT and CVC, D-dimer, Albumin, Platelet, and inflammatory response

	With UEDVT (n = 11)	Without UEDVT (n = 55)	P value
CVC placement			
+	0	7	0.21
−	11	48	
D-dimer			
< 0.5	0	0	*
≥ 0.5	9	14	
Non-measured	2	41	
Albumin	2.682	2.678	0.98
Platelet	24.74	20.62	0.0818
CRP levels, median (range) (mg/dl)	16.9	14.3	0.29
WBC levels, median (range) (/μl)	8.07	8.5	0.69

*CVC* central venous catheter, *CRP* C-reactive protein, *WBC* white blood cell

*The number of < 0.5 is zero, Chi-square analysis is impossible

## Discussion

LEDVT is well-known, but the frequency of UEDVT is not negligible due to the characteristic of esophagectomy, which conducts cervical manipulation and reconstructs through a narrow retrosternal pathway [11]. According to the American College of Surgeons National Surgical Quality Improvement Program Participant Use Data File (ACS-NSQIP PUF), the incidence of DVT is about 7%, and UEDVT comprises 57% of all DVTs in the analysis of more than 460,000 cases of general surgery, except esophagectomy [15]. It is reported that primary UEDVT is only about 20%, and its leading causes are venous thoracic outlet syndrome, Paget-Schroetter syndrome, and idiopathic, while secondary UEDVT accounts for 80% [16]. The predominant cause of secondary UEDVT is a CVC placement, which occupies at least two-thirds, followed by cancer and major surgery within 30 days [7]. Malignancy has been reported to increase the risk of UEDVT in 18-fold [17], and the presence of CVC scored with an odds ratio of 9.7(CI = 7.8–12.2) [18]. In our present study, the incidence of UEDVT was 16.7% in 66 consecutive patients who underwent esophagectomy with retrosternal gastric tube reconstruction, which is less frequent than the previous report that the incidence of UEDVT was 25.5% in retrosternal gastric tube reconstruction [11].

**Fig. 3:**
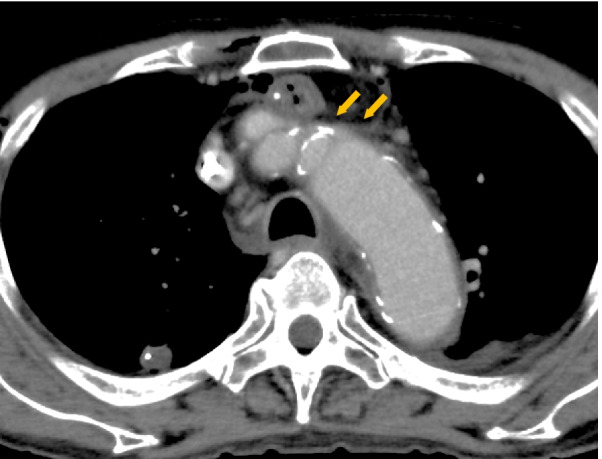
Non-UEDVT case after esophagectomy with gastric tube through the retrosternal reconstruction route. Open arrows indicate the compression of the left brachiocephalic vein by gastric tube and brachiocephalic artery

Several reports mentioned the disadvantages of the retrosternal route, such as cardiac compression and reconstructed organs’ necrosis, when the retrosternal space is narrow [19, 20]. Retrosternal reconstruction has been reported as an independent risk factor for UEDVT after esophagectomy, and its ratio of UEDVT is 25.5%, significantly higher than 4.9% of posterior mediastinal reconstruction [11]. Retrosternally shifted gastric tube may contribute to the left brachiocephalic vein’s compression and form UEDVT in the proximal left-side veins (Fig. 3). In the present study, all UEDVT occurred in the proximal left-side veins. Anastomotic leakage, inflammatory response, and other factors did not show any significant differences in generating UEDVT. Among several factors in the reconstructive route, the width of retrosternal space has been mentioned as an important factor affecting compression strength [11]. Following the definition of the retrosternal space ratio, our cutoff value (0.21) [AUC: 0.92 (95% CI: 0.84–0.99)] shows high sensitivity of 87% and specificity of 82%, which is similar to the previous report.

Considering the incidence of UEDVT, posterior mediastinal reconstruction should be preferable if the retrosternal width is less than 0.21. But when it comes to other complications, such as anastomotic leakage, especially in the high-risk group, retrosternal reconstruction might be better to deal with. Depending on each case background, we should consider the reconstruction route individually.

It has been widely believed that CVC placement is the most frequent reason for generating UEDVT in various disease and gastrointestinal cancer patients, but no study has described the relationship between CVC and UEDVT in esophagectomy cases. Our present study revealed no significant difference to cause UEDVT whether CVC exists or not. The frequency of UEDVT without CVC cases was 18.6% and is comparable to the previous report [11]. Since this case series consisted of a minor population of patients receiving CVC insertion, it is difficult to draw a solid conclusion regarding if the CVC insertion could increase the incidence of UEDVT in esophagectomy patients, and further study will be required. However, this is the first report to describe whether the presence of CVC affects the UEDVT’s formation in patients who underwent esophagectomy with retrosternal reconstruction in the current clinical practice.

For the prevention of DVT, the guideline recommends a variety of approaches as prophylaxis, such as Low Molecular Weight Heparin (LMWH) and compression stockings, but there are few references with regard to UEDVT [21, 22]. In all our cases, LMWH was administered subcutaneously as postoperative thromboprophylaxis, but the frequency of UEDVT was almost the same as previously reported.

Moreover, according to the Antithrombotic Therapy and Prevention of Thrombosis, 9th edition Guideline, 3 months of continuation of anticoagulant therapy with LMWH, vitamin K antagonists, and factor Xa inhibitor is recommended for UEDVT. In our cases, we prescribed edoxaban, a factor Xa inhibitor and their UEDVTs were disappeared in all but two cases [23]. As previous studies have reported that the mortality was significantly higher in the group of UEDVT without anticoagulants, it might be preferable to continue oral anticoagulant therapy if the risk of bleeding is low [24, 25].

As a diagnostic tool, CT scan has high sensitivity and specificity with 91% and 93%, respectively [26], whereas ultrasonography has high sensitivity (97%) and specificity (96%) [26], however still controversial regarding clinical probability and objectivity [27]. D-dimer’s sensitivity and specificity at the cutoff value of 500 μg/L were 92–100% and 14–60%, respectively, hence the accuracy of specificity remains to be discussed [10, 28]. In our study, D-dimer’s elevation did not show specificity by itself. Taken together, we believed that CT scan in the early period after esophagectomy might be superior to the other diagnostic tools.

Limitations of this study are the retrospective data nature, the small number of patients at a single institute. Comparison of usefulness for prevention with or without anticoagulation was not validated. It remains that all the UEDVTs that developed after the 4th postoperative day might be missed. CT scan might also be decision-making information for UEDVT occurrence during admission before completing prophylactic anticoagulation. However, since this is the first study to describe the evaluation of UEDVT with or without CVC placement and postoperative thromboprophylaxis after esophagectomy, we believe that the present study will provide useful information to clinicians, and further accumulation of retrospective and prospective multi-institution studies is required.

In conclusion, the incidence of UEDVT is not rare in patients who underwent esophagectomy with retrosternal reconstruction, but unlike other diseases, the width of retrosternal space, not the existence of CVC, is responsible for the development of UEDVT.

## Data Availability

The datasets generated and/or analyzed during the current study are not publicly available due to the protection of personal information of the patients, but are available from the corresponding author on reasonable request.

## Abbreviations

UEDVT: Upper extremity deep vein thrombosis
PE: Pulmonary embolism
CVC: Central venous catheter
LMWH: Low-molecular-weight-heparin
CT: Computed tomography
PICCs: Peripherally inserted central catheters
